# SOPAR: Bee Localization and Tracking Using RF Sub-harmonic Oscillating Parametric Resonator in Hive Environments

**DOI:** 10.1145/3810192

**Published:** 2026-06-15

**Authors:** QIJUN WANG, PEIHAO YAN, GEO JIE ZHOU, XIANG LIU, DAN STANLEY, ZACH HUANG, CHUNQI QIAN, HUACHENG ZENG

**Affiliations:** Michigan State University, USA

**Keywords:** Bee, localization, tracking, backscatter tag, RF sensing, sub-harmonic oscillator, parametric resonator

## Abstract

Honey bees play a vital role in global agriculture. Understanding the movement of a queen bee within a hive box is essential for advancing both biological research and practical apiculture. In this paper, we present SOPAR, a novel Radio Frequency (RF) Sub-harmonic Oscillating Parametric Resonator designed for non-invasive localization and tracking of a queen bee inside a full-size hive box. The core component of SOPAR is a lightweight RF backscatter tag that can be attached to a bee’s thorax without affecting its normal behavior. The tag comprises two passive resonators: (i) an inner spiral inductor bridged by a varactor diode and (ii) an outer circular inductor with a gap bridged by a chip capacitor. The outer inductor harvests energy from the external excitation signal, driving the oscillation of the inner spiral resonator to produce sub-harmonic backscattered signals at half the excitation frequency. The frequency separation between excitation and backscatter signals eliminates self-interference at the RF reader, significantly improving signal-to-noise ratio (SNR) and detection range. The layout of the two resonators is meticulously optimized to maximize magnetic coupling, thereby minimizing the overall tag size. Building on this dual-resonator tag, we design an RF reader with a Bayesian estimation algorithm that localizes the tagged bee by exploiting the spatio-temporal characteristics of the sub-harmonic backscattered signals. We have built a prototype of SOPAR, featuring a tag with a diameter of only 3.7 mm and a weight of less than 10 mg. Extensive experiments demonstrate that SOPAR achieves a median localization error of 3.7 cm when tracking a queen bee in a full-size hive box. Moreover, the results confirm that SOPAR remains robust under diverse environmental conditions.

## 1 INTRODUCTION

Honey bees (Apis mellifera) play an indispensable role in global agriculture as they are the most important pollinators among all insects and animals. In the United States alone, crops dependent on honey bee pollination are estimated to contribute over $15 billion annually to the agricultural economy [45]. A bee colony is typically housed in a hive box containing multiple combs, each composed of hexagonal wax cells that serve as the structural foundation for egg laying, brood rearing, and food storage. Within this highly organized social structure, the queen bee is the sole reproductive individual. Her primary role is to maintain colony growth by laying 2,000–3,000 eggs per day, while her movement patterns govern the spatial and temporal dynamics of colony activity. Tracking the queen’s movement within a hive provides critical insights into colony behavior and health. For example, how long does a queen remain on a single comb? How does her movement vary across seasons or environmental conditions? Beyond egg-laying, does the queen patrol the hive when not ovipositing? Addressing these questions could greatly enhance our understanding of social coordination in honey bee colonies and enable practical advances in beekeeping, such as improved queen management, early detection of colony disorders, and optimization of hive productivity.

However, tracking the movement of a bee in a beehive environment is challenging. Most existing behavioral studies rely on cameras in observation hives using computer vision techniques [7, 18, 31, 36, 42, 44, 49]. When using a camera to track bees within a hive, the hive box typically contains only one to four vertically arranged combs enclosed by transparent sidewalls for visual monitoring. This type of hive does not represent the three-dimensional structure of a standard colony and cannot capture natural queen dynamics within the dark interior of a full-size hive box. Recent advances have shown that RF signals can support sensing tasks beyond communication, including fine-grained behavioral and physiological monitoring in challenging environments [22, 51-53, 58, 59]. RF-based tracking technologies have also emerged as a promising alternative for insect localization [1, 3, 24, 25]. In these systems, a miniature RF tag is attached to an insect’s thorax, and an external RF reader detects the signal backscattered from the tag for localization and motion tracking. This wireless approach has the potential to operate without optical visibility and can be integrated into beehives without structural modification. However, existing RF-based efforts have remained limited to simulations or controlled in vitro measurements, as the size and weight of typical tags make them unsuitable for in vivo attachment on real bees. Moreover, most existing tags rely on linear resonant backscattering, where the weak return signal occurs at the same frequency as the strong excitation signal, leading to severe self-interference and drastically limiting detection range.

In this paper, we present SOPAR, a novel RF backscatter system that enables continuous and non-intrusive tracking of a bee within a hive box. Unlike prior harmonic or same-frequency backscatter systems, SOPAR introduces a subharmonic parametric backscatter mechanism that changes both the tag’s operating mechanism and the localization observable. The system comprises an RF backscatter tag attached to the bee’s thorax and an RF reader positioned outside the hive for signal excitation and detection. Designing SOPAR involves addressing several key challenges:

- **Challenge #1: Miniaturization and lightweight design.** The tag must be significantly smaller and lighter than the bee’s body to avoid affecting its natural behavior. Ideally, the tag weight should be no more than 5% of the bee’s body weight [2, 32, 39, 54]. For a worker or queen bee, the tag mass should remain below a few tens of milligrams while maintaining mechanical robustness and electromagnetic performance.
- **Challenge #2: Low-frequency operation for signal penetration.** Although high-frequency RF signals provide finer localization accuracy compared to low-frequency signals, they are severely attenuated by the honey- and wax-filled frames within a beehive box. Therefore, the system must use low-frequency signals (e.g., less than 2 GHz) to ensure sufficient penetration through dense structural materials and thus enable consistent detection throughout the entire hive volume. However, designing an efficient backscatter tag whose size is orders of magnitude smaller than the wavelength of its excitation frequency remains a major challenge.
- **Challenge #3: Self-interference suppression.** Conventional backscatter systems suffer from interference between the excitation and reflected signals due to frequency overlap. Achieving frequency separation (e.g., by generating a harmonic or frequency-shifted backscatter) can substantially improve signal-to-noise ratio (SNR) and extend the detection range. This requirement adds another layer of challenge in the design.

SOPAR addresses these challenges through a co-design of the backscatter tag and the RF reader. To minimize the tag’s size and weight, the backscatter tag adopts a dual-resonator architecture. The outer resonator is a circular inductor that harvests energy from the external excitation field, while the inner resonator is a spiral inductor that is driven by the harvested energy to generate backscatter signals. The two resonators couple magnetically, rather than electrically, with the reader antenna. Unlike electric coupling, which typically requires the tag’s dimensions to approach half the wavelength of the excitation signal, magnetic coupling eliminates this constraint, enabling a much smaller form factor suitable for bee attachment. To mitigate the self-interference issue, SOPAR introduces a nonlinear capacitor in the inner spiral inductor and a fixed capacitor in the outer circular inductor. Specifically, the spiral resonator is concentrically enclosed within the circular resonator, which locally concentrates magnetic flux at the excitation frequency. The terminal edges of the spiral inductor are bridged by a varactor diode, whose voltage-dependent capacitance induces sub-harmonic oscillation and backscattering at half the excitation frequency. The separation of excitation and backscatter frequencies fundamentally addresses the self-interference issue for the RF reader, significantly improving its detection reliability and range.

The integration of spiral and circular resonators within a single concentric structure enables both frequency separation and energy harvesting while not requiring active electronic components or an on-tag power source. The layout of the two resonators is carefully optimized to not only maximize the energy collection from the incident excitation field but also amplify their local magnetic flux, significantly improving the backscattering efficiency. This configuration enhances power transfer efficiency while maintaining a compact form factor. Our rigorous model-based analysis confirms that the backscattered signal indeed occurs at half the excitation frequency and that the required activation voltage for backscattering is an order of magnitude lower than that of conventional RF tags. For a hyper-abrupt varactor diode with a junction potential of 0.7 V and a grading coefficient of 0.7, the required pumping voltage across the varactor diode is the inverse of the resonator’s quality factor (i.e., 1∕Q). For a resonator with Q=33, the required pumping voltage is only 0.03 V. In contrast, generating a DC voltage using a conventional power-harvesting antenna and rectifier requires a threshold voltage of roughly 0.7 V. Therefore, the proposed resonator achieves an activation voltage an order of magnitude lower than traditional RF energy-harvesting systems.

SOPAR’s RF reader serves as the excitation source and the receiver for backscattered signals, enabling continuous localization of tagged bees. During operation, the RF reader transmits an excitation wave to activate the passive backscatter tag and simultaneously monitors the reflected signals to estimate the tag’s position. The localization algorithm leverages a key empirical observation: the tag’s response is highly sensitive to the excitation power. When the incident signal strength falls below a certain threshold, the tag becomes inactive and ceases to backscatter. This nonlinear activation behavior provides a distinct boundary between active and inactive regions, which can be exploited for spatial localization. Based on this observation, SOPAR employs a spatio-temporal activation strategy to determine the bee’s position within a beehive box. Spatially, an array of antenna elements is distributed along the outer surfaces of the hive box to provide directional coverage. Temporally, the RF reader transmits a single-frequency, amplitude-swept continuous wave as the excitation signal. By jointly controlling the excitation amplitude and the active antenna element, the RF reader can probe different regions of the hive box and infer the bee’s location from the activation pattern of the tag. This method effectively overcomes the limited detection range of individual antennas, enabling full-volume tracking coverage of an entire beehive box.

We have conducted in vivo experiments using live bees placed within beehive boxes to evaluate the performance of SOPAR under realistic conditions. The RF reader and antenna array were mounted externally on the hive walls, while tagged bees freely moved inside the comb-filled structure. During these trials, the tagged queen bee exhibited continuous frame-to-frame traversal and normal comb engagement patterns, with no observable restriction in locomotion or access to hive structures. Extensive experimental results show that SOPAR achieves a median localization error of 3.7 cm when tracking a queen bee in a full-size managed hive box. Furthermore, the results confirm that SOPAR remains robust across diverse environmental conditions. Table 1 summarizes a comparison between SOPAR and existing approaches in the literature.

The main contributions of this work are summarized as follows.

- It presents the first bee-scale batteryless sub-harmonic parametric backscatter tag, introducing device-level frequency separation between excitation and backscattered signals.
- It develops an end-to-end and low-cost bee tracking system capable of localizing a queen bee within a beehive box, enabling new opportunities in biological research and practical apiculture.
- It demonstrates system effectiveness through in vivo experiments with real bees, showing superior detection reliability, localization coverage, and robustness compared to existing solutions.

## 2 PROBLEM DESCRIPTION

The queen bee plays a central role in maintaining the stability and productivity of a honey bee colony. Her presence, fertility, and movement patterns directly influence colony health, reproduction, and overall hive behavior. Tracking the queen bee’s location provides critical insights into colony dynamics, such as brood distribution, swarm prediction, and environmental stress responses. However, most existing tracking methods rely on manual inspection or optical observation, which are labor-intensive, intrusive, and often unreliable inside natural hive environments. Automated, non-invasive tracking of the queen bee enables more precise monitoring, offering beekeepers and researchers a valuable tool for improving hive management and understanding collective insect behavior.

**Fig. 1.**
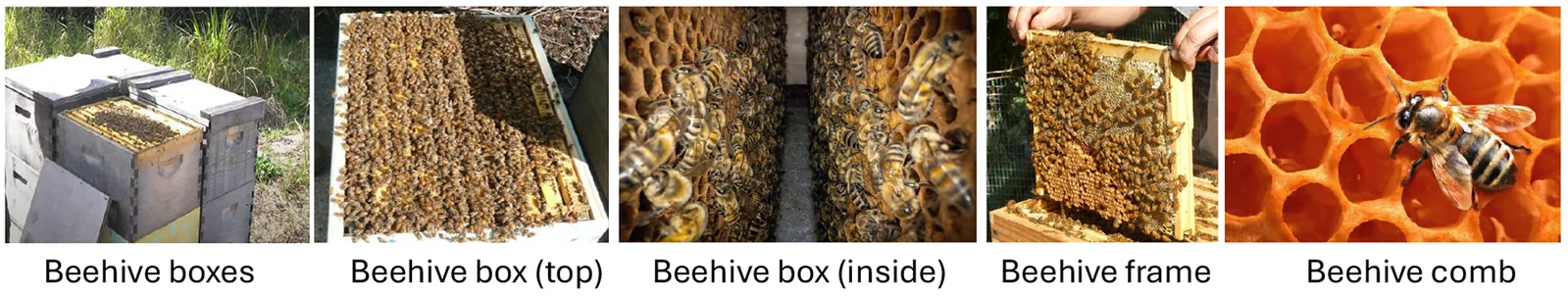
Illustration of the beehive structure and composition.

As shown in Fig. 1, a natural or managed beehive consists of a dense arrangement of comb frames made of beeswax, filled with honey, pollen, larvae, and adult bees. These wax and honey-filled structures create a complex, lossy medium that significantly absorbs and scatters radio waves, particularly at high frequencies. The hive box itself is typically constructed from wood or other dielectric materials, further contributing to signal attenuation. Inside this environment, thousands of bees are in constant motion, continuously altering the electromagnetic propagation paths. This intricate biological and structural composition makes it challenging to maintain consistent wireless communication and signal detection for an electronic tag placed within the beehive.

Developing a reliable RF-based queen bee tracking system presents several key challenges. First, the small size and limited payload capacity of the bee impose strict constraints on the tag’s weight, power consumption, and antenna dimensions, making conventional active tracking devices unsuitable. Second, RF signal propagation within a beehive is severely attenuated and distorted due to the honey, wax, and biological tissues, reducing both communication range and localization accuracy. Third, backscatter-based localization systems must contend with strong self-interference between excitation and reflected signals, as well as undesired harmonic distortion from RF components. Finally, achieving accurate localization in such a cluttered and dynamic environment requires precise signal processing and robust frequency design. Overcoming these challenges demands an innovative RF tag architecture and a reader system capable of operating effectively within the unique electromagnetic conditions of a natural or managed beehive.

## 3 TAG DESIGN: PARAMETRIC RESONATOR

### 3.1 Design Considerations and Choices

Some key system parameters must be meticulously selected to strike a balance between hardware constraints and localization performance. In the beehive environment, space and weight limitations are critical, as the tags must be small and lightweight enough not to disturb the bees’ natural behavior. At the same time, the system must maintain sufficient localization accuracy despite the complex, cluttered, and dynamic environment inside the hive.

#### Excitation Frequency

For localization, higher excitation frequencies generally provide finer spatial resolution because the shorter wavelength enables more precise estimation of the signal phase and amplitude variations. However, a natural beehive is densely packed with combs of wax and honey, both of which exhibit high dielectric loss and cause significant attenuation of electromagnetic waves, especially at higher frequencies. To ensure reliable signal penetration while maintaining reasonable localization precision, SOPAR selects an excitation frequency at about 1.5 GHz, striking a balance between propagation robustness and localization accuracy within the hive environment.

#### Tag Resonance Type

The RF coupling between the RF reader and the backscatter tag can occur through either electric coupling or magnetic coupling. Electric coupling relies on capacitive effects and typically requires the tag’s physical dimension to approach half the excitation wavelength, which is unsuitable for miniaturized designs. In contrast, magnetic coupling transfers energy via near-field inductive interaction and does not impose a strict wavelength-dependent size constraint. In addition, the loop-based magnetic resonator provides a smoother angular response than dipole-like electric radiators, so orientation primarily scales coupling strength rather than introducing sharp nulls in the link. Therefore, SOPAR employs magnetic coupling by designing a compact circular resonator as the base of the tag. This resonator magnetically couples with the reader’s antenna to harvest energy from the excitation field and re-radiate it as a backscattered signal.

#### Self Interference

While a single circular resonator can generate backscattered signals, it reflects them at the same frequency as the excitation wave, leading to a severe self-interference problem. Because the backscattered signal is orders of magnitude weaker than the transmitted excitation signal, it becomes difficult for the RF reader to detect it amidst strong leakage and reflection. To overcome this limitation, one widely used approach is to convert the excitation signal to its harmonic frequency (i.e., $2f_{\text{ex}}$ or $3f_{\text{ex}}$ with $f_{\text{ex}}$ being the frequency of the excitation signal), thereby creating frequency separation between the excitation and backscattered signals. This frequency translation allows the reader to filter out the strong excitation component and detect the much weaker backscattered signal more effectively.

However, this approach is not well-suited for bee tracking in beehives for two reasons. *First*, in practical RF systems, undesired harmonic components are inevitably generated by the nonlinearities of active circuit elements such as power amplifiers, mixers, and oscillators. As a result, when the RF reader transmits the excitation signal, it also produces harmonic frequencies that coexist with the desired fundamental tone. These unintended harmonics can fall within the spectral band of the backscattered signal, causing interference that degrades detection sensitivity and localization accuracy. *Second*, the doubled or tripled harmonic signals experience much stronger attenuation during propagation, particularly when penetrating the honey- and wax-filled comb structures inside the hive. The higher frequency components suffer greater dielectric loss and reduced penetration depth, significantly diminishing the detectable backscatter power. Consequently, harmonic backscattering is unsuitable for reliable operation in the highly attenuating environment of natural beehives.

To address the self-interference issue, SOPAR adopts a dual-resonator structure that achieves effective frequency separation while maintaining low power consumption and a compact form factor suitable for bee-mounted operation. Importantly, through this novel design, SOPAR realizes sub-harmonic backscattering, in which the tag re-emits a signal at half the excitation frequency. This mechanism not only provides clear spectral separation from the excitation wave but also avoids interference from undesired harmonics generated by the RF reader. Moreover, operating at a lower sub-harmonic frequency improves propagation through the dense, lossy materials of the beehive, enhancing both detection sensitivity and localization reliability.

### 3.2 Tag Architecture and Design Principles

#### Tag Architecture

**Fig. 2.**
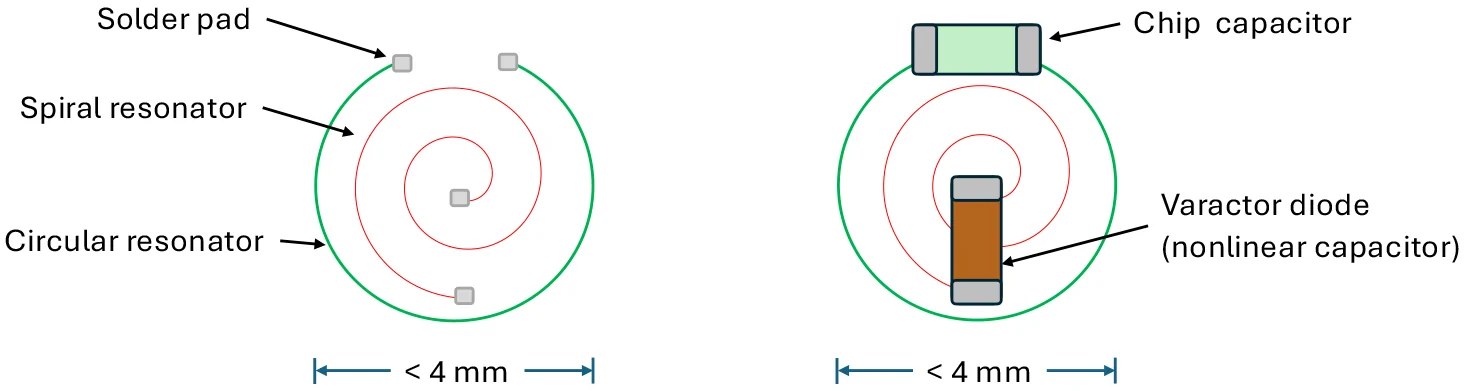
Schematic of the backscattering tag used for bee localization. Left: tracing diagram; right: complete circuit diagram.

The proposed RF tag features a dual-resonator architecture specifically engineered to generate backscattered signals at a sub-harmonic frequency of the excitation source. As illustrated in Fig. 2, the tag comprises two concentric resonant elements: (i) a nonlinear spiral resonator at the core, and (ii) an outer circular resonator that functions as an energy-harvesting and coupling enhancement structure. The spiral resonator is realized as a two-turn planar inductor whose terminal ends are connected through a varactor diode, forming a nonlinear LC tank circuit. This compact spiral exhibits a high quality factor, typically greater than 30, allowing efficient storage and exchange of electromagnetic energy at its natural resonance frequency $f_{0}$. To enable biological applications, the overall resonator size is minimized such that it can be mounted on a bee’s thorax without interfering with its normal movement or behavior.

Encircling the spiral resonator is a circular resonator, which acts as the power-harvesting loop. This element is carefully designed to capture energy from the external excitation field at the pumping frequency and to concentrate magnetic flux in the vicinity of the spiral structure. The circular loop is bridged with a chip capacitor, tuning it to resonate near the excitation frequency. Through close magnetic coupling between the two resonators, the energy harvested by the outer loop (circular inductor) is efficiently transferred to the inner spiral, driving its nonlinear oscillation. This concentric coupled-resonator configuration not only enhances magnetic coupling and power transfer efficiency but also maintains a highly compact footprint suitable for miniature and mobile sensing applications.

#### Sub-harmonic Backscattering

The spiral resonator generates a backscattered signal at half the frequency of its pumping signal, provided by the concentric circular inductor. This sub-harmonic response arises from the nonlinear behavior of the varactor diode bridging the spiral inductor. The varactor’s capacitance depends on the voltage across it. Under small-signal operation, the voltage-capacitance relation can be expressed as $C(t)=C_{0}{\left(1-\frac{V_{p}}{\phi }\right)}^{-\lambda }\approx C_{0}(1+\lambda \frac{V_{p}}{\phi })$ for small-signal operation, where $v_{p}$ is the excitation voltage induced across the varactor diode by the pumping signal, ϕ is the junction potential determined by the diode’s structure and materials (e.g., ϕ=0.7V), λ characterizes the abruptness of the junction, and $C_{0}$ is the zero-bias capacitance of the junction. This voltage-dependent capacitance introduces nonlinearity into the resonator, enabling frequency mixing processes that can transfer energy from the high-frequency pumping signal to a lower-frequency mode. When an external pumping signal $f_{p}$ is applied to the varactor diode, the nonlinear interactions within the varactor induce parametric oscillation. At the initial stage of oscillation, the spiral resonator is excited to generate two frequency components, $f_{b}$ and $f_{c}$, that satisfy $f_{b}+f_{c}=f_{p}$. As the oscillation evolves, the oscillating signals converge when their frequencies coincide, i.e., $f_{b}=f_{c}$. Under this degenerate condition, the spiral resonator oscillates at a single frequency $f_{0}=f_{p}∕2$, which is the observed sub-harmonic response.

#### Efficiency Analysis

From an energy perspective, the varactor within the spiral resonator converts a fraction of the pumping signal’s energy into the sub-harmonic frequency resonance mode. The conversion efficiency scales with the square of the capacitance modulation, i.e., ${\left(\lambda V_{p}∕\phi \right)}^{2}$. It competes against dissipative losses in the resonator, which scale as $\left(1∕Q^{2}\right)$ with Q being the resonator’s quality factor. When the pumping signal is sufficiently strong to compensate for these losses, sustained oscillation occurs at the sub-harmonic frequency $f_{0}$. The inclusion of the concentric power-harvesting resonator further enhances coupling to the pumping field. It ensures that sufficient energy reaches the nonlinear varactor diode within the spiral resonator to maintain this stable sub-harmonic oscillation.

The varactor diode within the spiral resonator requires a voltage exceeding a certain threshold to sustain its oscillation. This activation voltage threshold determines the tag’s detection range for a given RF reader transmission power. A lower threshold enables a longer detection range, and vice versa. Therefore, an important question arises: what is the minimum voltage across the varactor diode required to sustain oscillation for signal backscattering? To address this, we present the following theorem.

Theorem 3.1. *The minimum voltage required across the varactor diode within the spiral resonator to sustain parametric oscillation is given by* $V_{p}^{⋆}=\phi ∕(\lambda Q)$.

The proof of Theorem 3.1 is provided in the Appendix 7.

For a hyper-abrupt varactor diode, the junction potential ϕ≈0.7V and the grading coefficient λ≈0.7, making the required pumping voltage $V_{p}$ approximately equal to the inverse of the circuit’s quality factor 1∕Q. For a resonator of Q=33, the required pumping voltage is only 0.03 V. In contrast, generating a DC voltage using a conventional power-harvesting antenna and rectifier requires a threshold voltage of approximately ϕ=0.7V. Therefore, the proposed resonator exhibits an activation voltage that is an order of magnitude lower than that of conventional RF power harvesters. This ultra-low activation threshold is a direct consequence of parametric oscillation and forms the physical basis that enables SOPAR ’s miniaturization beyond the limits of rectifier-based or harmonic tags.

#### Design Innovations

Several innovations distinguish this sub-harmonic resonance tag from conventional backscatter or RFID devices. *First*, the integration of spiral and circular resonators within a single concentric architecture enables both frequency conversion and energy harvesting without requiring active electronic components or external power sources. The circular resonator not only collects energy from the incident excitation wave but also amplifies the local magnetic field, significantly improving the coupling to the spiral resonator. This configuration enhances power transfer efficiency while preserving a compact form factor. *Second*, the use of a varactor-loaded spiral resonator introduces a controlled nonlinearity that allows efficient parametric mixing and frequency division. Unlike linear RFID tags, which use on-off modulation, this architecture inherently performs frequency translation, producing backscattered signals at a sub-harmonic of the interrogation frequency. Such frequency separation minimizes interference between excitation and response signals, thereby simplifying receiver design and improving detection sensitivity. *Finally*, this design leads to an extreme miniaturization of the entire tag, making it feasible to be mounted on small biological platforms such as bees. The lightweight and passive nature of the system allows autonomous operation without disturbing natural behaviors. This combination of nonlinear parametric oscillation, dual-resonator coupling, and biologically compatible miniaturization represents a fundamental advance in the design of passive, batteryless sub-harmonic backscatter systems.

## 4 RF READER: A SPATIO-TEMPORAL LOCALIZATION ALGORITHM

### 4.1 Overview

#### System Setup

**Fig. 3.**
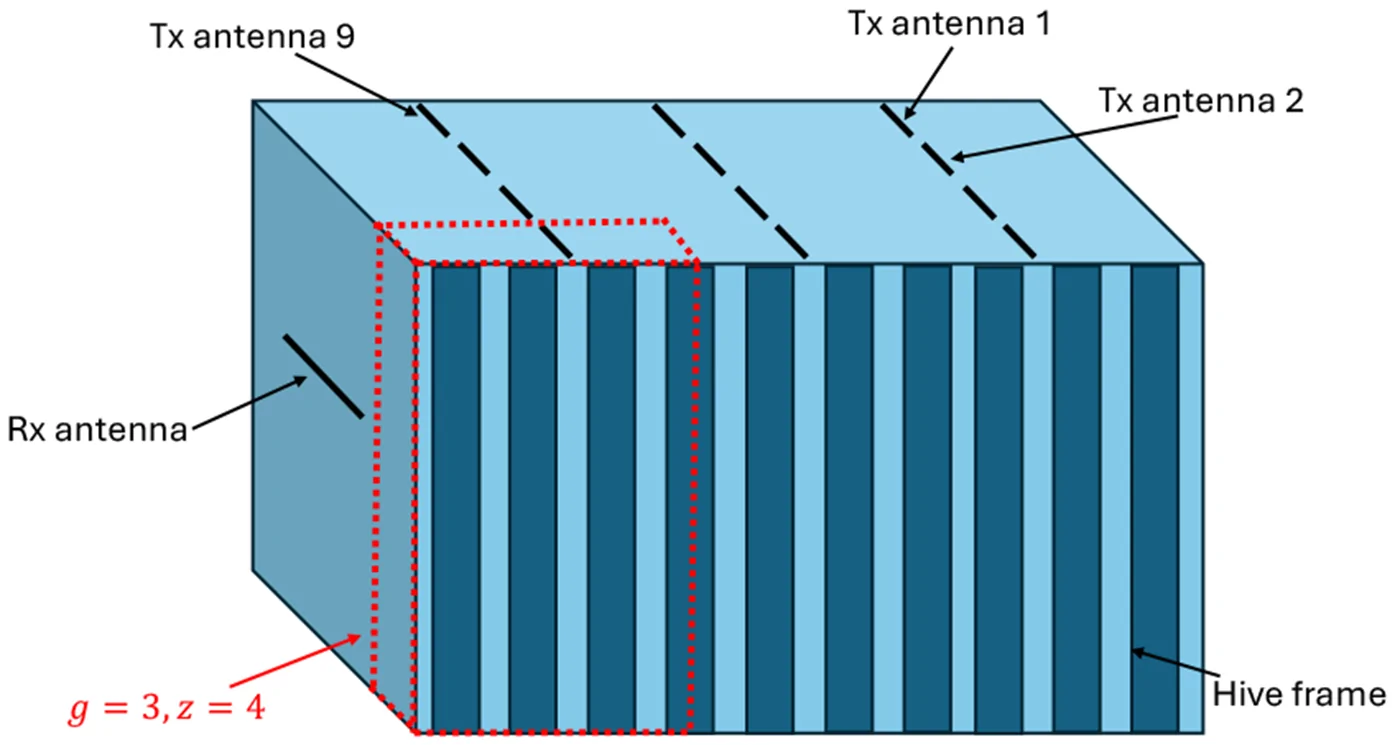
System setting. The beehive was constructed from wood and consisted of nine vertical frames. Twelve transmitting (Tx) antennas were mounted on the external surface of the hive box, and one receiving (Rx) antenna was attached to the side surface.

The backscatter tag is mounted on a bee that naturally moves within a hive, as shown in Fig. 3. To enable full-volume excitation and signal collection, we deploy an array of (M=12) transmit (Tx) antennas evenly distributed along the outer surface of the hive’s top side, while a single receive (Rx) antenna is placed on the outer surface of one hive side. All Tx antennas share the same RF chain through an RF switch, allowing the reader to activate one antenna at a time for controlled spatial excitation. A radio device, connected to both the RF switch and the Rx antenna, coordinates signal transmission and reception. This configuration ensures efficient energy delivery and signal coverage across the entire hive. It is worth noting that we use M transmit antennas instead of M receivers because the tag is a passive device that requires a certain amount of excitation energy to activate its sub-harmonic oscillation. By distributing multiple Tx antennas evenly across the hive’s surface, the system can generate more uniform excitation fields throughout the hive volume. This spatial diversity ensures that the tag, regardless of its position, is energized above its activation threshold. This setting enables robust detection and full localization coverage.

#### Spatio-Temporal Sweeping of Signal Transmission

The tag becomes active only when its received excitation power exceeds a specific threshold, which depends on the RF reader’s transmission power and the tag’s distance from the active Tx antenna. To exploit this nonlinear activation property, we propose a spatio-temporal sweeping algorithm for the excitation signal transmission. In the spatial domain, the RF reader sequentially activates one Tx antenna at a time through the RF switch following a given scheduling strategy. Since the tag may only respond under a subset of antennas, this selective activation pattern provides valuable spatial cues for localization. In the temporal domain, for each Tx antenna, the RF reader gradually increases its transmission power from zero to ($P_{\max}$) during a time slot. The tag activates only when the excitation signal power surpasses the threshold, leading to a distinct transition from inactive to active states. The changes in tag orientation mainly lead to small shifts in the activation threshold, which are naturally handled by the power sweep and multi-port fusion rather than causing missed detections. By measuring the ratio between inactive and active durations within the slot, the RF reader can infer the relative proximity of the tag with respect to those Tx antennas, enabling localization within the hive.

#### One-Time Calibration

Before deployment, one-time calibration is performed to capture the field patterns of a hive for fine-grained tag localization. The tag is placed at a sparse set of checkpoints covering corners and the center at different heights. At each point, the reader sweeps its antenna ports while ramping amplitude, recording stabilized descriptors that reflect signal strength and persistence. These measurements are aggregated, smoothed, and normalized to create a spatial template for each antenna port, indicating where it is likely to show strong activation. A dispersion and reliability value are also estimated for each port. This collection of templates is stored as a map, which is then used at runtime to compare observed signals and generate per-port likelihoods. These likelihoods are combined during fine localization and tracking within the grid defined by the coarse stage. The calibration only needs to be repeated when the hive geometry or antenna placement changes.

#### Signal Processing Pipeline

**Fig. 4.**
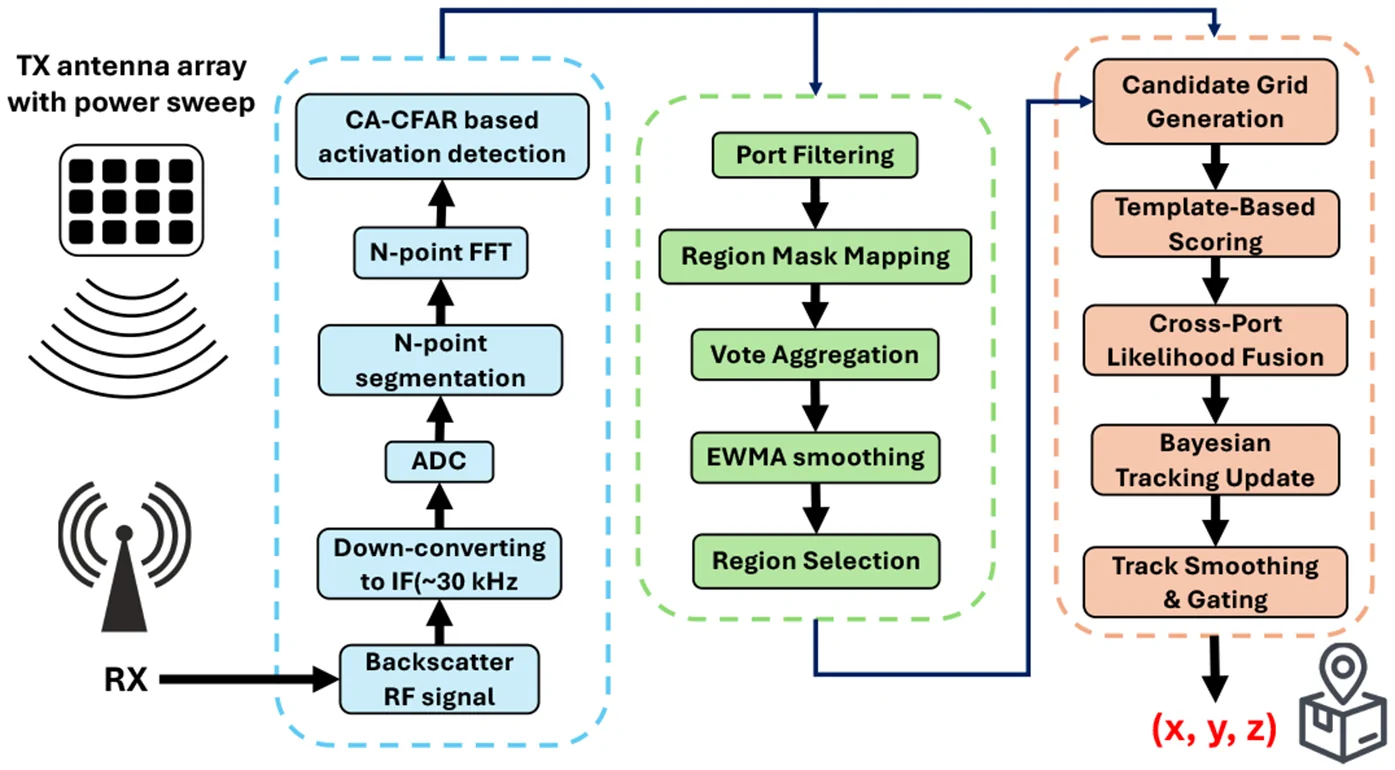
Signal processing pipeline.

**Fig. 5.**
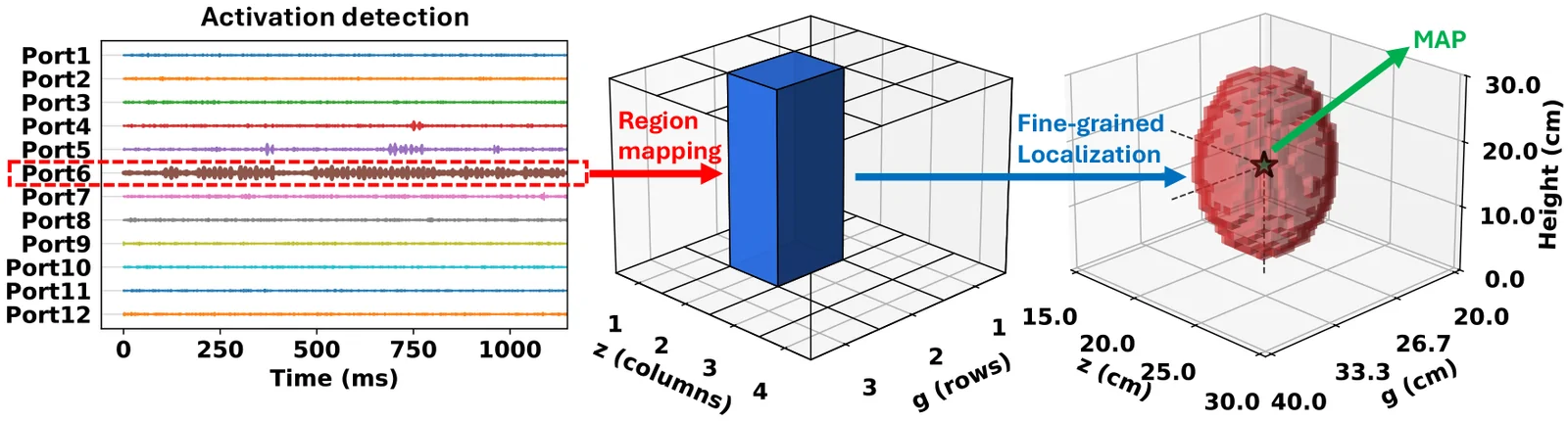
An instance that illustrates the localization procedure.

Based on the spatio-temporal signal sweeping, we propose the signal processing pipeline as shown in Fig. 4 to estimate the location of the tagged bee. The sub-harmonic signal received by the RF reader is first down-converted to an intermediate frequency (e.g., 30 kHz). Then, it is sampled and transformed by an n-point FFT, from which the tag’s reflection bin is selected. We then apply CA-CFAR (Cell-Averaging Constant False Alarm Rate) to decide whether the tag is “on” for that port. From each detected activation, we extract two intuitive features: *activation strength* (how far the peak rises above the threshold) and *activation duration* (how long the signal stays above threshold within the port’s time slot). Using these features, coarse localization performs a soft spatial vote to narrow the tag’s location to a likely region. Finally, we compare the observed per-port evidence against a pre-calibrated spatial response template and combine the likelihoods across all ports using a Bayesian fusion strategy, which produces a stable and precise 3D position within the selected region. Fig. 5 provides an example over a time window: it shows per-port time-domain activations, the resulting coarse search region, and the final fine-grained 3D estimate. In what follows, we explain the key modules of this pipeline in detail.

### 4.2 Power-Swept Activation Detection

**Fig. 6.**
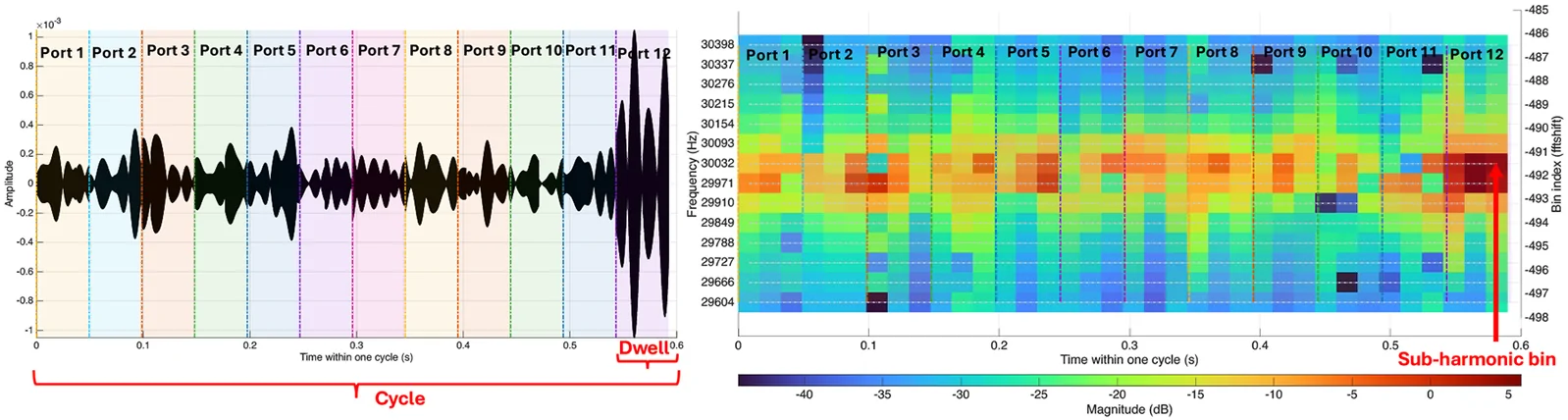
Per-cycle visualization combining time- and frequency-domain views.

Under an amplitude-swept single-tone excitation, each steering port, indexed by s, yields one steady dwell per scan cycle k. After discarding short head/tail guards to ensure steady state, we compute a narrowband spectrum around the calibrated sub-harmonic bin $b_{0}(s)$ using a short DFT/Goertzel window. Let $S_{s,k}[b]$ denote the *power*-spectrum (magnitude-squared) of the steady portion. To absorb minor oscillator and thermal drift, we take the signal statistic as the local peak $s_{\text{sig}}=\max_{|b-b_{0}|\leq \delta }S_{s,k}[b]$, where δ is a small bin tolerance (typically 1 ~ 2 bins). Tick-wise on/off decisions are made by a hysteretic CA-CFAR around the $b_{0}(s)$ neighborhood using thresholds ($\Theta_{\text{high}}$, $\Theta_{\text{low}}$). Here, s is the antenna port index, k is the scheduling cycle index, and b indexes the frequency bins of the dwell spectrum. Fig. 6 illustrates the signals in both time and frequency domains in a scheduled cycle.

#### CA-CFAR Baseline with Hysteresis

CA-CFAR is a widely used adaptive thresholding technique in radar systems that estimates the noise level by averaging surrounding reference cells to maintain a constant false alarm rate under varying clutter conditions. Here, we use it to estimate the tag’s activation timing when the RF reader performs power sweeping in a given time slot. Specifically, the activation threshold refers to the minimum received excitation level at which the tag enters the active backscattering state under a given port and dwell condition, whereas the activation boundary refers to the corresponding spatial separation between active and inactive regions induced by this threshold during the sweep. Accordingly, SOPAR exploits the threshold-crossing pattern across ports and cycles, rather than absolute signal strength alone, for downstream localization. We run CA-CFAR within each per-port dwell to form an adaptive hysteretic threshold at the sub-harmonic bin. We then detect on/off activation and derive the onset, offset, and on-duration for that slot.

A CA-CFAR baseline is estimated from side-bands separated by guard cells, yielding local mean/standard-deviation (μ, σ) and a hysteretic pair of adaptive thresholds

(1) $$\Theta_{\text{high}}=\mu +k_{\text{high}}\sigma ,\;\;\;\Theta_{\text{low}}=\mu +k_{\text{low}}\sigma ,$$

where $k_{\text{high}}$ and $k_{\text{low}}$ are fixed operating points from installation-time calibration. When hysteresis is disabled, we use $\Theta \equiv \Theta_{\text{high}}$.

#### Two Complementary Descriptors

We form two descriptors that are robust yet informative for downstream fusion.

- *Descriptor 1: Activation Margin*. Since $s_{\text{sig}}$ is in the power domain, we define

(2) $$\Delta_{\text{dB}}\;≜\;10\;\log_{10}(s_{\text{sig}})-10\;\log_{10}(\Theta_{\text{high}}),$$

which quantifies how strongly the sub-harmonic peak exceeds the adaptive CA-CFAR threshold on a logarithmic scale.

- *Descriptor 2: Activation Persistence.* Within the steady dwell, we evaluate $s_{\text{sig}}$ on a short sequence of analysis ticks, where a tick is one fixed-hop window advance that yields a single $s_{\text{sig}}$ sample. A tick is marked “on” if $s_{\text{sig}}>\Theta_{\text{high}}$ and remains “on” until $s_{\text{sig}}<\Theta_{\text{low}}$ (hysteresis). Let $r_{\text{act}}$ be the number of “on” ticks and $T_{\text{ref}}$ the total steady ticks; we use the normalized persistence

(3) $$p_{s,k}=\text{clip}\left(\frac{r_{\text{act}}}{T_{\text{ref}}},0,1\right).$$

which suppresses brief threshold crossings and captures how stably the tag stays active under the power ramp, complementing the instantaneous activation margin.

#### Instantaneous confidence and EWMA smoothing

A dwell is considered active if either $p_{s,k}\geq r_{0}$ or $\Delta_{\text{dB}}\geq \Delta_{0}$, where $\left(r_{0},\Delta_{0}\right)$ are conservative operating points determined by a short calibration sweep. For soft voting, we compress the two descriptors into a continuous *instantaneous* confidence ${\tilde{v}}_{s,k}\in (0,1)$ that increases with both dB margin and persistence:

(4) $$v_{s,k}=\text{sigm}\left(\beta \;(\Delta_{\text{dB}}-\Delta_{0})\right)\cdot p_{s,k}^{\gamma },$$

where $\text{sigm}(u)=\frac{1}{1+e^{-u}}$, parameter β>0 controls the steepness of the logistic mapping on the dB margin, and γ∈[0,1] modulates the influence of persistence (γ=1 gives a linear weight). To stabilize across cycles while remaining responsive to genuine onsets, we maintain the exponentially weighted moving averages (EWMAs) of the following features:

(5) $${\tilde{p}}_{s,k}=(1-\alpha ){\tilde{p}}_{s,k-1}+\alpha \;p_{s,k},\;\;\;{\tilde{\Delta }}_{s,k}=(1-\alpha ){\tilde{\Delta }}_{s,k-1}+\alpha \;\Delta_{\text{dB}},\;\;\;{\tilde{v}}_{s,k}=(1-\alpha ){\tilde{v}}_{s,k-1}+\alpha \;v_{s,k},$$

with a small forgetting factor α matched to the sweep period.

The per-port descriptors forwarded to the modules presented in Sec. 4.3 are $\left\{{\tilde{p}}_{s,k},{\tilde{\Delta }}_{s,k},{\tilde{v}}_{s,k}\right\}$ together with the hysteresis state. This interface preserves complementary evidence (persistence vs. amplitude margin), is robust to slow gain drift via CA-CFAR normalization, and keeps latency bounded by the dwell duration since all statistics are evaluated in-stream around $b_{0}(s)$ with a single local spectral evaluation per tick.

### 4.3 Coarse Localization: Spatio-Temporal Voting

We aggregate stabilized per-port descriptors into a compact search region indexed by a group g and, within that group, a longitudinal zone z. The red dashed region in Fig.3 marks an example with g=3, z=4.

#### Geometry

Rows of antenna elements mainly resolve across adjacent comb frames (hence the group label g), while columns resolve along the comb within a frame (hence the longitudinal zone z). Each antenna element (r, c) predominantly supports a spatial patch ${𝒲}_{r,c}\approx {ℱ}_{r}\times {ℒ}_{c}$. From installation-time sketches or a sparse calibration sweep, we derive *fixed* per-port soft masks $M_{s}(g)\in [0,1]$ and $M_{s}(g,z)\in [0,1]$ that satisfy $\sum_{g}M_{s}(g)=1$, and for fixed $g\;:\sum_{z}M_{s}(g,z)=M_{s}(g)$. Masks are obtained by sparse sampling of the sub-harmonic strength over representative locations and then smoothed (e.g., bi-/tri-linear) with mild Tikhonov regularization to avoid overfitting; they remain fixed during operation.

#### Spatial Reliability and Pooling

On cycle k, we use the stabilized descriptors $\left\{{\tilde{p}}_{s,k},{\tilde{\Delta }}_{s,k},{\tilde{v}}_{s,k}\right\}$ to form a reliability set ${𝒮}_{k}^{\text{on}}=\{s\;:{\tilde{p}}_{s,k}\geq p_{\text{min}}\;\wedge \;{\tilde{\Delta }}_{s,k}\geq \Delta_{\text{min}}\}$, and softly penalize ports outside it via binary weights

(6) ws,k={ωson,s∈𝒮kon,ωsoff,otherwise,}withωson≥1,ωsoff∈(0,1),

where $\omega_{s}^{\text{on}}$ and $\omega_{s}^{\text{off}}$ are port-dependent to reflect historical noise or coupling. We then compute *normalized* scores by weighted pooling of $v_{s,k}$ through the geometry masks:

(7) $$G_{k}(g)=\frac{\sum_{s}w_{s,k}{\tilde{v}}_{s,k}M_{s}(g)}{\sum_{s}w_{s,k}M_{s}(g)+\varepsilon },\;\;Z_{k}(g,z)=\frac{\sum_{s}w_{s,k}{\tilde{v}}_{s,k}M_{s}(g,z)}{\sum_{s}w_{s,k}M_{s}(g,z)+\varepsilon },$$

where a tiny ε avoids division by zero for regions with negligible coverage.

#### Temporal Smoothing and Selection

To reduce jitter while preserving true motion, we apply EWMAs with forgetting factors $\left(\lambda_{G},\lambda_{Z}\right)$ matched to the scan period, obtaining ${\tilde{G}}_{k}(g)$ and ${\tilde{Z}}_{k}(g,z)$. We then select the coarse region in two steps: $g^{⋆}=\text{arg}\;\max_{g}{\tilde{G}}_{k}(g)$, $z^{⋆}=\text{arg}\;\max_{z}{\tilde{Z}}_{k}(g^{⋆},z)$. We track selection margins $\Delta_{G}={\tilde{G}}_{k}(g^{⋆})-\max_{g\neq g^{⋆}}{\tilde{G}}_{k}(g)$ and $\Delta_{Z}={\tilde{Z}}_{k}(g^{⋆},z^{⋆})-\max_{z\neq z^{⋆}}{\tilde{Z}}_{k}(g^{⋆},z)$ and apply a simple hysteresis: when $\Delta_{G}<\tau_{G}$, keep the previous $g^{⋆}$ unless a challenger exceeds it by at least $\tau_{G}^{\prime }$ for two consecutive cycles; an analogous rule with ($\tau_{Z}$, $\tau_{Z}^{\prime }$) is used for $z^{⋆}$. These guards operate at the region level and are independent of the dwell-level hysteresis in Sec. 4.2.

#### Confidences

Smoothed scores are converted to normalized confidences via temperature-scaled softmax:

(8) $$P_{k}(g)=\frac{\text{exp}(\eta_{G}{\tilde{G}}_{k}(g))}{\sum_{g^{\prime }}\text{exp}(\eta_{G}{\tilde{G}}_{k}(g^{\prime }))},\;\;\;P_{k}(z\;|\;g^{⋆})=\frac{\text{exp}(\eta_{Z}{\tilde{Z}}_{k}(g^{⋆},z))}{\sum_{z^{\prime }}\text{exp}(\eta_{Z}{\tilde{Z}}_{k}(g^{⋆},z^{\prime }))}.$$

The selected region is ($g^{⋆}$, $z^{⋆}$); the tuple $\left(\Delta_{G},\Delta_{Z},P_{k}(g),P_{k}(z\;|\;g^{⋆})\right)$) quantifies selection strength and is carried forward.

### 4.4 Fine Localization & Tracking: Bayesian Fusion over a Constrained Search Region

We estimate a continuous 3D state $x_{k}\in R^{3}$ on a fine grid restricted to the region indexed by ($g^{⋆},z^{⋆}$) with a small spatial buffer. When the zone margin $\Delta_{Z}$ is small, the adjacent runner-up zone is included as well. Denote the (possibly anisotropic) grid by ${𝒳}_{k}={\left\{x_{i}\right\}}_{i=1}^{N_{k}}$. The observation at cycle k is the stabilized per-port (Tx antenna) descriptor set ${\left\{{\tilde{v}}_{s,k}\right\}}_{s=1}^{S}$ constructed in Sec. 4.2. The coarse-stage quantities $\left\{P_{k}(g),P_{k}(z\;|\;g^{⋆}),\Delta_{G},\Delta_{Z}\right\}$ are only used to constrain ${𝒳}_{k}$ and to modulate the process noise.

#### Per-port Spatial Templates and Likelihood

For each steering antenna port s, we use a precomputed spatial template $\pi_{s}(x)\in [0,1]$ from installation-time calibration, representing the propensity that port s exhibits strong activation at location x (near-field coverage and multipath are implicitly absorbed; the map is smoothed and clipped). Given ${\tilde{v}}_{s,k}\in (0,1)$, we adopt a bounded Gaussian error model $\ell_{s}(x_{i})=\text{exp}\left(-\frac{\eta_{s}}{2\sigma_{s}^{2}}{\left({\tilde{v}}_{s,k}-\pi_{s}(x_{i})\right)}^{2}\right)$, with dispersion $\sigma_{s}^{2}$ from calibration (or a warm-up sweep) and reliability $\eta_{s}\geq 0$ (set $\eta_{s}=0$ for missing/muted ports and reduce it for historically noisy ones). Assuming conditional independence across ports gives the joint likelihood ${ℒ}_{k}(x_{i})=\prod_{s=1}^{S}\ell_{s}(x_{i})$. In practice, environmental variations inside the hive may cause a mismatch with the calibrated spatial templates, appearing as consistently low or spatially inconsistent likelihoods across ports. This behavior can be monitored online as a reliability indicator. Re-calibration is needed when the hive shape or antenna placement changes. In such cases, recalibration can be performed using a small set of anchor points without removing bees or disassembling the hive.

#### Bayesian Update on Constrained Grid

The posterior over ${𝒳}_{k}$ follows

(9) $$p_{k}(x_{i})=\frac{{ℒ}_{k}(x_{i})\;p_{k|k-1}(x_{i})}{\sum_{j=1}^{N_{k}}{ℒ}_{k}(x_{j})\;p_{k|k-1}(x_{j})},\;\;\;x_{i}\in {𝒳}_{k},$$

and $p_{k}(x_{i})=0$ for $x_{i}\notin {𝒳}_{k}$. When $\Delta_{Z}$ is below a permissive threshold, we widen ${𝒳}_{k}$ to include the runner-up zone *before* normalization to avoid premature commitment near boundaries.

#### Motion Prediction (Process Model)

Let ${\hat{x}}_{k-1}$ be the refined center from the previous cycle (defined below), ${\hat{v}}_{k-1}$ an exponentially smoothed velocity, and $\Delta_{t}$ the scan period. We use a near-constant-velocity prior centered at ${\hat{x}}_{k}^{-}={\hat{x}}_{k-1}+{\hat{v}}_{k-1}\Delta t$ with anisotropic diffusion

(10) $$p_{k|k-1}(x_{i})\propto \text{exp}\left(-\frac{1}{2}{\left(x_{i}-{\hat{x}}_{k}^{-}\right)}^{T}\Sigma_{\text{proc}}^{-1}\left(x_{i}-{\hat{x}}_{k}^{-}\right)\right),\;\;\Sigma_{\text{proc}}=\text{diag}(\sigma_{∥}^{2},\sigma_{⊥}^{2},\sigma_{z}^{2}),$$

where diffusion is larger along physically plausible directions (e.g., within-frame) and smaller orthogonally; the overall scale shrinks as the coarse confidence $P_{k}(g^{⋆})P_{k}(z\;|\;g^{⋆})$ increases and gently expands when it is low, improving recovery from occasional misvotes. The velocity is updated by ${\hat{v}}_{k}=(1-\rho ){\hat{v}}_{k-1}+\rho \;\left({\hat{x}}_{k}-{\hat{x}}_{k-1}\right)∕\Delta t$ with a small ρ.

#### State Extraction and Sub-grid Refinement

We take the MAP index $i^{⋆}=\text{arg}\;\max_{i}p_{k}(x_{i})$ and refine to sub-grid precision by a local quadratic fit of the log-posterior over the 3 × 3 × 3 neighborhood $𝒩(i^{⋆})$. Fitting $q(\Delta x)=c+b^{T}\Delta x+\frac{1}{2}\Delta x^{T}H\;\Delta x$ to ${\left\{\log\;p_{k}(x_{j})\right\}}_{x_{j}\in 𝒩(i^{⋆})}$ (least squares) yields ${\hat{x}}_{k}=x_{i^{⋆}}-H^{-1}b$ and $\Sigma_{k}=H^{-1}$, with $\Sigma_{k}$ clipped to remain positive definite. If $x_{i^{*}}$ is near the edge of ${𝒳}_{k}$ or H is poorly conditioned, we expand $𝒩(i^{⋆})$ by one cell per side and refit once; if still ill-conditioned, we fall back to the grid MAP $x_{i^{⋆}}$.

#### Template Construction and Calibration

Templates $\pi_{s}(x)$ are built from a sparse set of anchor poses and interpolated (e.g., trilinear with mild Tikhonov regularization) before clipping to [0, 1]. Dispersion $\sigma_{s}^{2}$ comes from early measurements of $\left({\tilde{v}}_{s,k}-\pi_{s}(\pi )\right)$ at known locations. The same calibration provides initial $\eta_{s}$ and anisotropy ratios in $\Sigma_{\text{proc}}$.

## 5 EXPERIMENTAL EVALUATION

### 5.1 Implementation

#### RF Tag

**Fig. 7.**
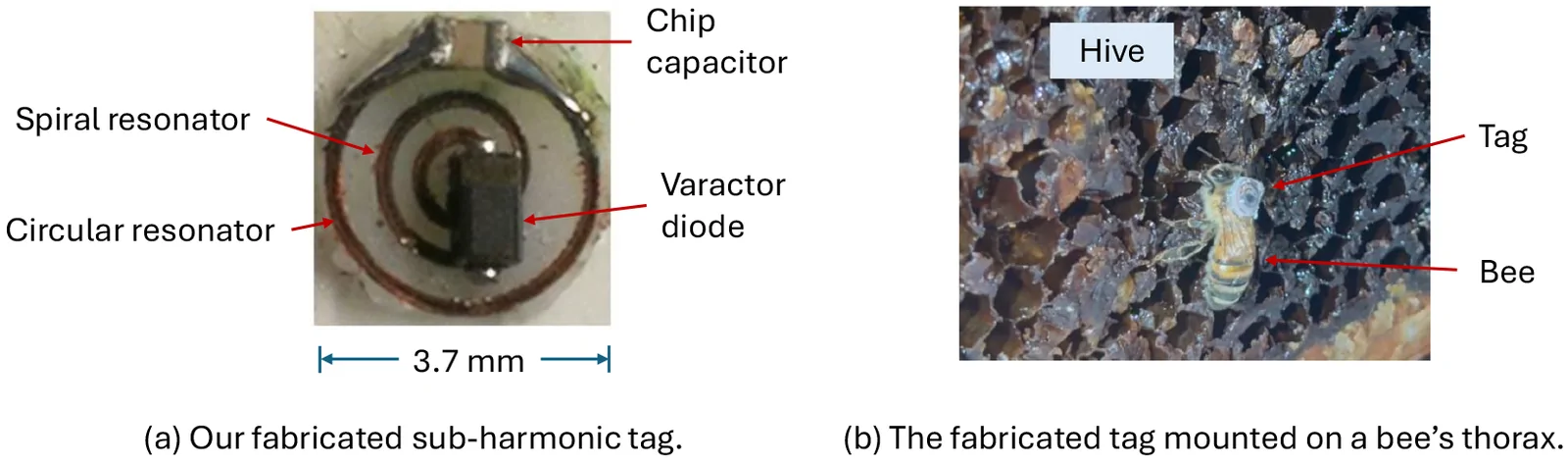
Our fabricated sub-harmonic tag for the bee’s localization in a hive box.

Fig. 7 shows a picture of our fabricated tag. The inner spiral resonator is a two-turn planar inductor, with a diameter 2.8 mm, trace width 0.3 mm, and inter-turn spacing 0.3 mm). Its terminal edges are bridged by a BBY51 varactor diode (Infineon), forming a nonlinear LC tank that resonates at 688 MHz with Q=34 when measured stand-alone. Surrounding the spiral resonator is the circular resonator, whose diameter is 3.7 mm and trace width is 0.3 mm. The circular resonator is split by a gap, which is bridged with a 2.4 pF chip capacitor. It is magnetically coupled to the spiral. The pair exhibits 672 MHz (Q=33) and 1344 MHz (Q=63) modes. This layout concentrates the pump field and strengthens coupling to the sub-harmonic mode with an extremely small footprint (≤ 4 mm in diameter). The total mass of the tag is about 10 mg, approximately 5% of the body weight of a queen bee. The tag is mounted on a queen bee’s thorax during the experiments.

#### RF Reader

**Fig. 8.**
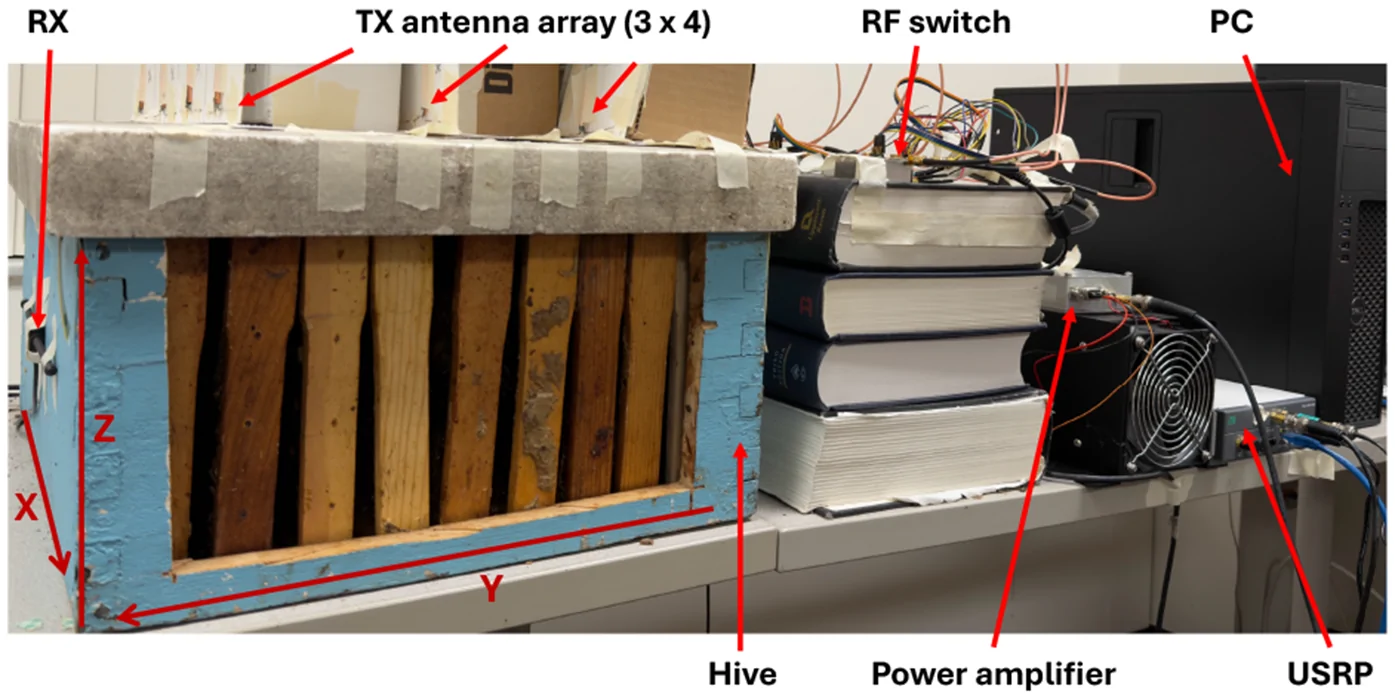
A picture of system components.

Fig. 8 shows our experimental setup. The hive is of a standard size, with 60 cm in length, 60 cm in width, and 30 cm in height. We define a hive-centered 3D coordinate system for localization, where the x-axis corresponds to the width (horizontal direction along a frame), the y-axis corresponds to the length (depth across frames), and the z-axis corresponds to the height (vertical direction). The origin is located at the lower-rear-right corner of the hive. The hive has nine frames. A 3 × 4 antenna array is attached to the top of the hive for signal transmission. One dipole antenna is attached to the hive’s side surface. A USRP N210, a power amplifier (PA), an RF switch, and a PC are used for signal transmission and reception. These components are used as a research instrumentation platform for flexibility. In practice, SOPAR only requires single-tone continuous-wave generation, programmable power sweeping, sequential RF switching, and narrowband sub-harmonic detection. These functions can be realized by a compact embedded RF reader using a fixed oscillator, PA, RF switch, signal detector, ADC, and microcontroller on a custom PCB, with an estimated total hardware cost below $30. The excitation signal is set to 1344 MHz. When powered by the excitation signal, the backscatter tag produces backscattered signals at about 672 MHz. They can be set to other frequencies by tuning the tag parameters. The entire signal-processing pipeline is implemented using GNU Radio on the PC for real-time localization and tracking.

#### Experimental Setting

The reader emits a single-tone signal and sweeps its amplitude while switching the Tx antenna ports to probe different regions of the hive box. Our evaluation covers both static checkpoints and mobile trajectories. For static tests, we first marked locations that span multiple frames and heights, placed the tagged bee at each location, and recorded estimates to compute axis-wise errors and the three-dimensional error. For moving tests, the bee traverses routes that move across frames and heights while we log synchronized ground truth and estimates to report trajectory fidelity and median error. Each location or path is repeated multiple times. We aggregate results across runs and report medians and percentiles as summary statistics rather than single trials. During our experiments, we obtained the ground-truth location data as follows. We record video using a fixed camera placed inside the hive and align the video frames with the RF data based on their timestamps. We manually annotated the video frames in which the tagged bee appears and measured the bee’s location relative to the known hive and frame dimensions. We then convert the bee’s image coordinates to the RF coordinate system, denoted as ($x_{gt}$, $y_{gt}$, $z_{gt}$), which serves as the ground truth.

#### IRB Approval

The experimental activities have been approved by the institution of the authors.

### 5.2 Sub-harmonic Backscatter Spectrum

**Fig. 9.**
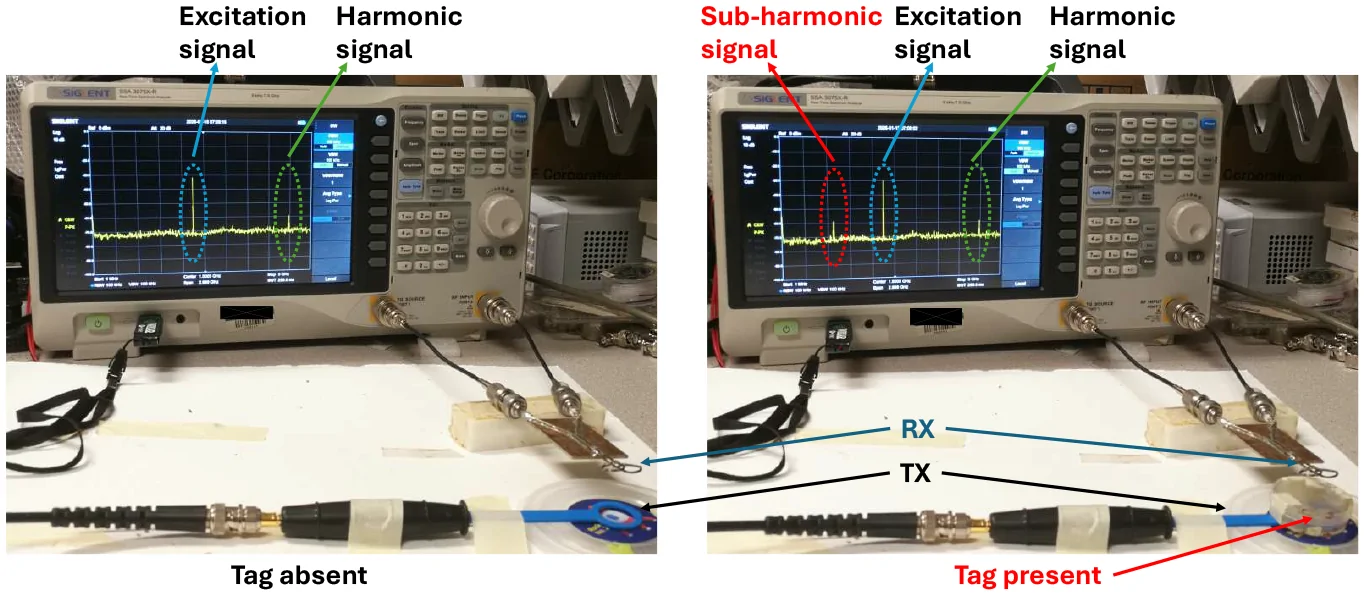
Measured RF spectra under two conditions: tag absent and tag present.

To verify the tag’s sub-harmonic operation, we measure the RF spectra under two conditions: with the tag absent and present. Fig. 9 shows our experimental setup. It can be seen that, when the tag is absent, only the excitation tone is observed together with weak higher-order harmonics (e.g., $2f_{\text{ex}}$). These harmonics arise from the inherent nonlinearity of RF instrumentation and active components. No spectral component appears near $f_{\text{ex}}∕2$. When the tag is present, in addition to the excitation tone and the same weak instrumentation-induced harmonics, a clear and stable component emerges at $f_{\text{ex}}∕2$. This component was generated by the tag’s nonlinear parametric oscillation. This confirms that the tag works as expected, generating sub-harmonic backscatter signals when excited. This also confirms that the tag achieves the desired frequency separation between excitation and reflection signals.

### 5.3 Case Studies

**Fig. 10.**
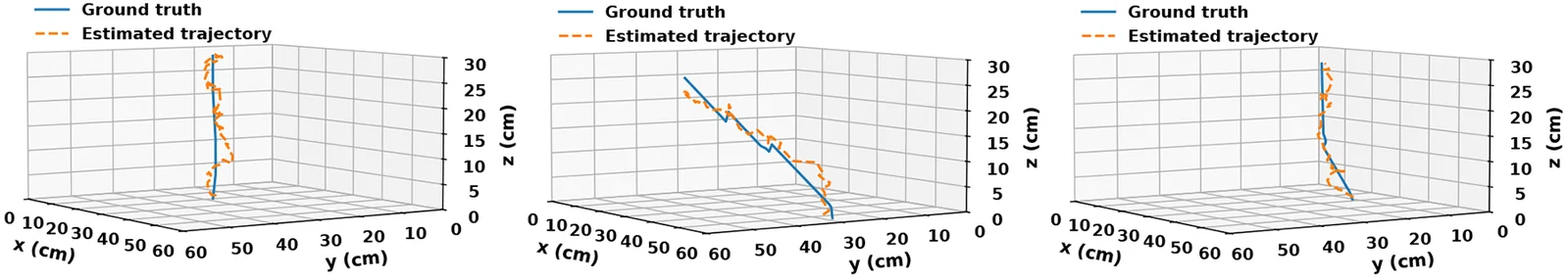
Estimated and ground-truth trajectories of a queen bee when it moves in a hive box.

To scrutinize the performance of SOPAR in tracking the movement of a tagged bee, we designed a trajectory study with common in-hive bee movements and report full 3D paths. The trajectories are plotted in the hive coordinate system defined in Sec. 5.1. The bee moves continuously through the hive across its frames and heights, either slowly or abruptly. We collect synchronized ground truth and estimate the tag traversed paths in three cases: (i) the bee moves from bottom to top, (ii) the bee travels from one corner to the opposite corner, and (iii) the bee follows straight and curved routes within the hive box. The durations of the three trajectories are 76 s, 176 s, and 136 s, respectively. These trajectories span multiple frames and cover different depths (front-back positions within a frame) and heights (vertical positions within a frame, from bottom to top). Fig. 10 visualizes the bee tracking results in these three cases. Across these runs, the estimated trajectories closely follow the ground truth and preserve overall shape, indicating that our localization remains reliable under diverse motion profiles.

In general, we observe that the tracking error reduces as the run proceeds. This is expected because the reader and estimator require a brief transient to stabilize. Specifically, the activation threshold settles after the first few amplitude sweeps, and the recursive smoother gains informative history to reduce localization errors. Once these states converge, the estimate aligns more tightly with the ground truth. Quantitatively, the median 3D errors for the three trajectories in Fig. 10 from left to right are 2.25 cm, 2.93 cm, and 2.33 cm, respectively.

### 5.4 Localization Accuracy

Since the bee is not always actively moving, we evaluate localization accuracy under both static and mobile conditions. We first create a 3D grid of pre-marked checkpoints that span multiple frames and heights inside the hive box. In the static case, we place the tagged bee at each checkpoint and record estimates. In the mobile case, the bee moves naturally and passes near these checkpoints while we log time-aligned estimates and ground truth. We compute absolute errors along x, y, z, and the combined three-dimensional error to understand which axis contributes more.

**Fig. 11.**
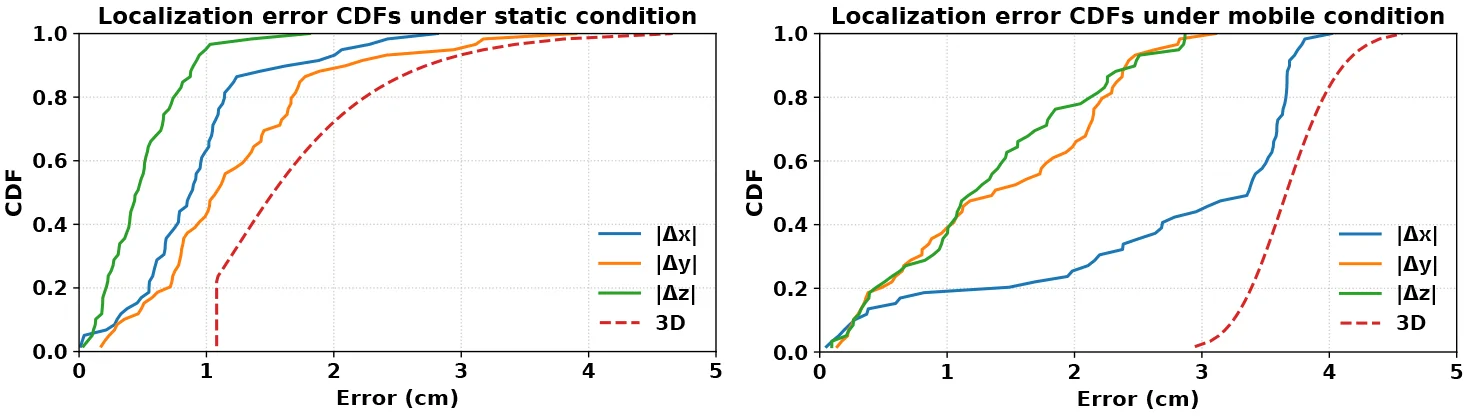
The measured localization errors along x, y, and z axes, as well as the absolute distance error in 3D space.

Fig. 11 shows the localization errors in the static and moving conditions. It can be observed that the system achieves better accuracy when the bee is static. This is expected. When the reflection tone and activation threshold remain stable, the reader collects more consistent samples at a single location, which improves the localization accuracy. Additionally, the recursive smoother benefits from stationary observations. It improves the signal-to-noise ratio and reduces the localization error. Overall, the median three-dimensional error is 1.54 cm for static checkpoints.

For mobile checkpoints, the measured median three-dimensional error is 3.67 cm. Axis-wise analysis shows that x contributes a significant error. This aligns with the typical motion pattern in which the bee travels within the same frame and moves from the outer region toward the inner region, which is largely along the x-axis. During a port sweep, the system samples ports in an order that is effectively aligned with x, so there is a small time skew across the measurements that are fused into a single position estimate. While the bee advances along x during this sweep, the fusion step mixes slightly outdated and current observations, which inflates x error more than y or z. Despite these effects, the mobile accuracy remains within a few centimeters, which is sufficient for continuous tracking inside the hive.

### 5.5 Tag-to-RX Distance

**Fig. 12.**
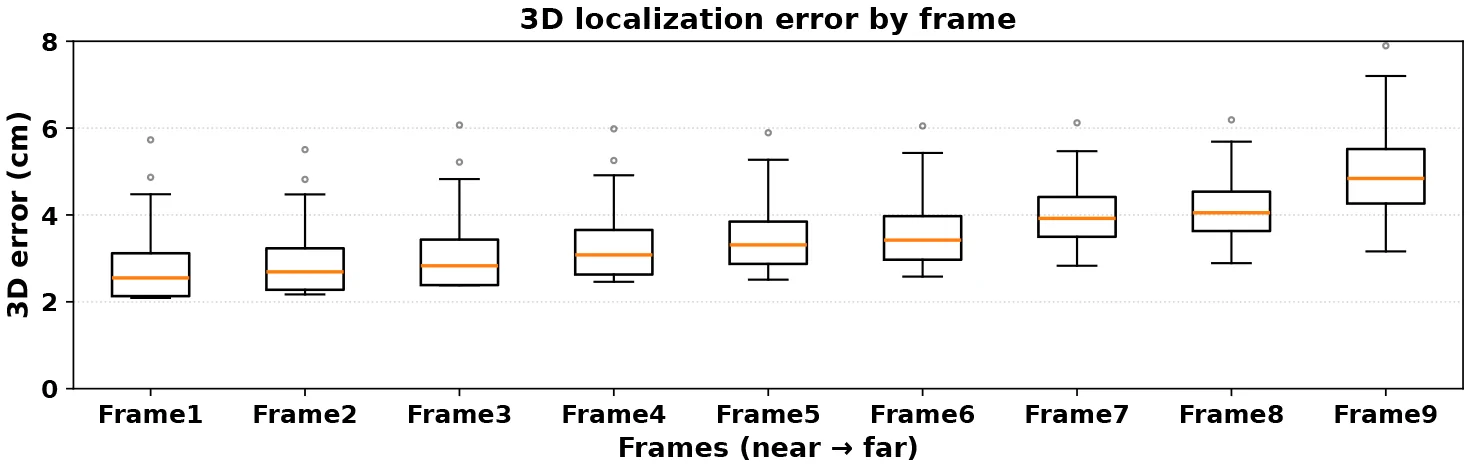
The measured absolute location error versus the frame index (i.e., tag-to-RX distance).

Unlike the transmit side that uses a 3×4 antenna array for spatial scanning, SOPAR employs a single Rx antenna mounted on the hive wall. We therefore study how the tag-to-Rx distance affects detectability and localization error across depth. Specifically, we place the tagged bee on frames one through nine, which progress from near to far relative to the RX antenna. Fig. 12 reports the errors per frame. The error generally increases as the bee moves farther from the RX antenna. This is expected because the backscattered signal attenuates with distance. The sub-harmonic component experiences additional loss when traveling through the comb and wax. As a result, the effective signal-to-noise ratio drops. Geometry also plays a role. Since the single RX antenna sits on one wall, depth is less observable than lateral directions. So small timing or amplitude variations across the TX antenna sweeping amplify into a larger depth uncertainty as distance increases. Hive materials can further introduce absorption and multipath that become more pronounced along deeper paths, inflating the error on the distant frames.

Despite these factors, the system maintains an acceptable localization accuracy across the entire depth range. The median localization error in 3D space over all frames is 3.42 cm, and the near-to-far trend is monotonic with only minor variations that likely stem from local frame geometry and material differences. These results indicate that SOPAR remains effective even when the bee is several frames away from the RX antenna. This highlights the benefit of placing the RX antenna closer to the expected region of bee activities in practice.

### 5.6 Robustness to Hive’s External Environments

We now study SOPAR’s robustness to the hive box’s external environments. To do so, we place different objects surrounding the hive and measure the localization accuracy under four conditions: (i) another hive box next to the target hive, (ii) several metal tools, (iii) a bucket of water, and (iv) a baseline with nothing placed beside the hive. All objects are positioned outside the hive along the side wall where the RX antenna is mounted, at a distance of approximately 2–5 cm from the hive surface, so that any perturbation to the magnetic coupling region would directly affect reception. The neighboring hive box has the same size as the target hive, the metal tools consist of common steel hand tools covering roughly a 30 cm × 25 cm area, and the water condition uses a plastic bucket of approximately 42 cm diameter filled with water. These configurations try to mimic the realistic beekeeping and workshop environments. Such objects may perturb the magnetic near field that SOPAR uses for wireless energy transfer and sub-harmonic backscatter. If that’s the case, they may raise the tag activation threshold and thus increase the localization error.

**Fig. 13.**
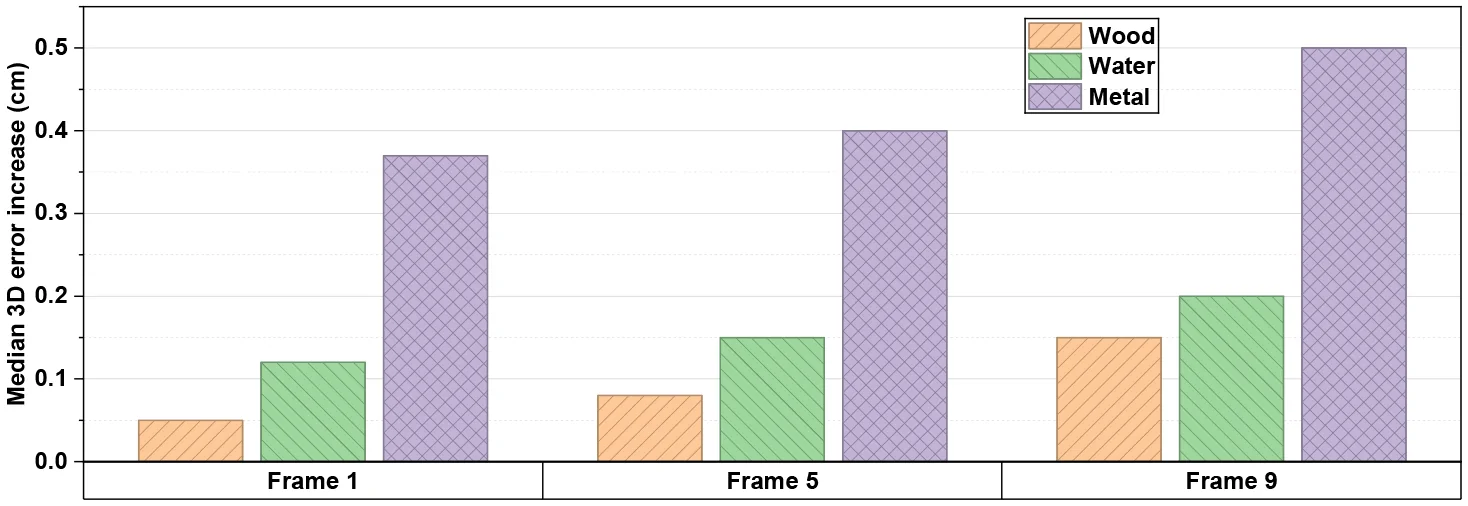
SOPAR’s robustness to external environments surrounding the target hive box.

Fig. 13 presents our experimental results. The bars report the increase in median 3D localization error relative to the baseline condition for Frames 1, 5, and 9, whose baseline median errors are 2.6 cm, 3.37 cm, and 4.83 cm, respectively. The measurements show a slight change in localization accuracy when a wooden hive box or a bucket of water is placed next to the target hive. Wood is a low-loss, low-conductivity material with a relative permittivity similar to that of dry organic matter, so it introduces minimal damping to the predominantly magnetic coupling between the reader and the tag. The bucket of water increases the electric field absorption. However, because our link primarily depends on magnetic coupling at short range, and the bucket lies outside the strongest coupling zone, its effect remains small. In contrast, placing metal tools near the hive produces a modest increase in error. This is because conductive objects support eddy currents that distort magnetic field lines, detune the coupling, and introduce reflections. These effects make the frequency offset estimation noisier. Even so, the degradation is slight for the following reasons: (i) the antenna array’s multiport excitation provides redundancy, (ii) the activation sweep adapts the threshold to local field variations, and (iii) the recursive smoother down-weights inconsistent samples. The experimental results confirm the robustness of SOPAR to the external environment of the target hive.

### 5.7 Robustness to Hive’s Internal Conditions

**Fig. 14.**
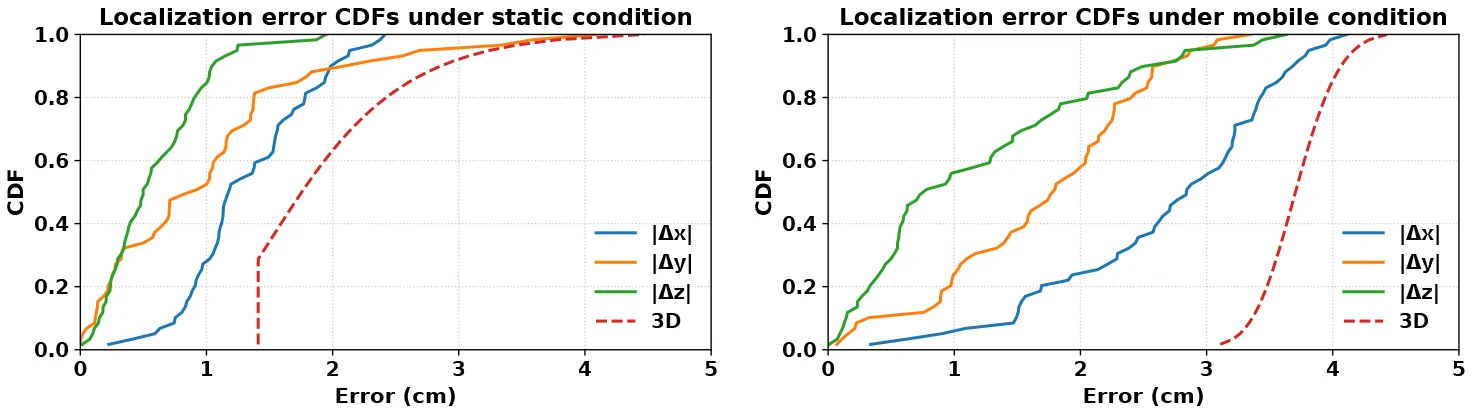
SOPAR’s robustness to the target hive box’s internal conditions.

To assess SOPAR’s robustness to hives’ internal conditions, we repeated the study using several different hive boxes that varied in frame occupancy, comb loading, and moisture level, reflecting natural variations across real hives. These factors may affect the signal propagation inside the hive because wax and honey introduce loss to the radio wave. Fig. 14 presents our measurement results for both static checkpoints and mobile passes near those checkpoints. It can be seen that the performance remains consistent across hive boxes. The median three-dimensional error is 1.77 cm for static checkpoints and 3.71 cm for mobile passes, similar to the results presented in the previous sections. There are two properties that support this stability. First, the link relies mainly on magnetic coupling at a short range, which is less sensitive to distant dielectric variations than purely electric field paths. Second, multi-port excitation provides spatial diversity. When local geometry or moisture slightly alters one coupling path, other ports still contribute reliable measurements that the smoother fuses over time. These results indicate that SOPAR is reliable across the hive’s diverse internal conditions.

## 6 RELATED WORK

### 6.1 Bee and Other Insect Tracking

#### Computer Vision Approach

Vision systems track bees either at hive entrances or inside observation hives. Entrance-facing cameras use stereo/depth cues to recover 3D flight paths in real time but remain sensitive to illumination and crowding on the flight board [8]. Inside observation hives, large-scale identification pipelines (e.g., BeesBook [5]) follow all marked individuals over weeks by learning correspondences across tag detections, enabling colony-level social analytics. Markerless [7] models push further by segmenting and tracking every bee without tags on natural comb backgrounds—achieving near body-width positional accuracy—yet long-term identity through heavy occlusion remains challenging [6]. Lightweight fiducials such as BEEtag [10] offer low-cost identity for bumblebees and honeybees, though persistence depends on view geometry and detection quality. Recent deep-learning pipelines (e.g., YOLO+MOT variants) track individuals inside hives and count or classify in/out traffic at entrances under outdoor lighting, but accuracy degrades under severe shadows, rain, and dense overlaps [48].

#### RF-based localization

Prior work spans passive RFID, custom backscatter, harmonic radar, BLE/active radios, and mmWave radar, each balancing tag form factor, communication range, and localization accuracy. A widely used bio-logging constraint is the “5% rule”: an effective tag should be ≤~ 5% of the animal’s body mass to minimize behavioral impact [2, 39]. For honey bees this implies milligram-scale tags (≈ 5–6 mg for workers; ≈ 9–11 mg for queens).

Ultra-miniature *UHF/HF RFID* tags enable identity and entrance/exit logging on honeybees and queens, e.g., a 25 mg queen-bee UHF tag at 868 MHz [25], 5–5.4 mg UHF/HF designs [2, 11, 47], and a heavier 81 mg UHF prototype on bumblebees with ~0.75 m read range [4]. These systems are attractive for batteryless operation and low mass (5–25 mg), yet linear backscatter at the same carrier is prone to strong reader self-interference and typically supports binary detection (presence, passage) rather than continuous in-hive localization.

Custom *backscatter* pushes range with larger payloads: *μ*Locate reports 60 m with ~1.45 m accuracy using multiband backscatter [35], and Living IoT reaches 80 m with ~1.9 m accuracy on bumblebees at 915 MHz [16]; both inherit self-interference from linear backscatter and rely on centimeter-scale antennas that increase tag mass/area. *Harmonic radar* mitigates self-interference via frequency-shifted returns and delivers long-range field tracking (5–800 m) across hornets, bumblebees, and melon flies with very light tags (≈0.8–15 mg), but accuracy is meter-scale (1.5–7 m) and transponders are centimeter-length, which limits in-hive deployment [30, 33, 34, 55]. *BLE/active radios* sacrifice batterylessness for telemetry and kilometer-class links (e.g., airdropped sensors to 1 km on moths [15] and ~160 m beetle platforms [14, 17]), with tag masses in the hundreds of milligrams to grams and thus higher tag-to-body ratios. Beyond these modalities, a honey-bee system at 5.8 GHz performs *bearing estimation via power-based AoA* using an energy-harvesting (piezoelectric) transmitter and a mechanically scanned patch array, achieving about ±5° angular error [41]. For migration-scale studies, active 915 MHz nodes (mSAIL) achieve LOS/NLOS ranges of 150/494 m and reconstruct tens of kilometers of trajectories for monarchs with a 62 mg package [20]. Finally, *77 GHz FMCW* radar attains ~0.3 m 3D accuracy over ~1.8 m using a 50 mg reflector on bumblebees [12], offering camera-free tracking but within short ranges and controlled volumes. Overall, reported tag-to-body mass ratios in the table span ~5–120%, highlighting the central trade-off: lighter, batteryless tags (HF/UHF RFID, harmonic transponders) favor detection with limited spatial precision, whereas longer-range or higher-accuracy methods (custom backscatter, BLE/active, mmWave, or power-based AoA setups) demand heavier tags and careful interference management.

Table 1 summarizes RF-based insect-tracking systems and situates SOPAR as a *batteryless*, millimeter-scale *subharmonic backscatter* design (TX/RX 1344/672 MHz) that suppresses reader self-interference and enables fine-grained in-hive localization with lower tag-to-body mass ratios than active/BLE approaches while maintaining practical ranges.

### 6.2 RF Tag Localization

A broad line of work customizes battery-free or ultra–low-power RF tags to trade between accuracy, latency, and deployability in cluttered spaces [9, 13, 21, 23, 26, 29, 40, 43, 56]—but most assumptions fail inside full-sized hives (dark, occluded, lossy media, tight geometry). *Nonlinear backscatter* separates uplink/downlink at a harmonic to suppress self-interference and unlocks wideband ranging, reaching ~3.5 cm 3D accuracy in controlled multi-static setups [27]; however, it assumes stable channels and clear apertures that are rare within dense combs. *FastLoc* fuses analog backscatter with structured-light cues to achieve 0.7 cm and sub-ms latency [50], but its optical path (and lighting) must remain line-of-sight—hard to guarantee under persistent occlusion. *MiNav* co-designs mmWave FMCW infrastructure with passive backscatter anchors for autonomous navigation and ~9 cm median error [19]; the required antenna apertures and clearance are mismatched with hive interiors. *RFly* extends passive UHF RFID range via drone relays and attains ~19 cm accuracy [28], yet depends on free-space mobility and line-of-sight access.

Active–assist designs using compact *UWB* transmit tags (e.g., Harmonium) deliver decimeter-scale real-time tracking via band-stitched reception [37], but GHz-range signals attenuate strongly in wax/honey, and multi-anchor synchronization is impractical in hives. Ultra–low-power *UWB backscatter* (Slocalization) integrates over long windows to approach sub-10 cm accuracy for static tags [38], trading responsiveness and relying on high-frequency propagation that fares poorly through hive materials. *OFDMA backscatter* enables batch localization with commodity-like readers [46], but decimeter precision still assumes sparse multipath and open layouts. Classic RF phase/motion–assisted methods (e.g., Tagoram) reach high precision with COTS RFID [57], while requiring known/induced trajectories and generous baselines. Overall, these studies illuminate levers—frequency shifting, wideband ranging, cross-technology cues, relays—but also why a hive-specific path is needed: strong high-frequency attenuation, severe occlusion, and strict size/power limits motivate SOPAR ’s subharmonic, magnetically coupled tag with reader co-design for in-hive operation.

## 7 CONCLUDING REMARKS

In this work, we presented SOPAR, a sub-harmonic oscillating RF resonator system for non-invasive localization and tracking of a queen bee within a full-sized hive. SOPAR introduces a millimeter-scale, lightweight inductive tag that operates through magnetic coupling and sub-harmonic backscattering, thereby eliminating self-interference and extending the effective detection range. By co-designing the tag architecture and multi-antenna reader, SOPAR achieves reliable localization in the dense, lossy environment of a beehive without requiring structural modification or optical access. Our experimental evaluation demonstrates that SOPAR provides an order-of-magnitude improvement in detection distance over existing bee-tracking sensors while maintaining robustness against environmental variations, and its ultra-low activation voltage further points to the potential for even smaller or more sensitive passive tags in future biological and environmental sensing applications.

While SOPAR demonstrates strong promise as a practical tool for in-hive queen localization, several limitations and scope considerations warrant discussion. With centimeter-level localization in a full-size, comb-filled hive, SOPAR can already support downstream analyses such as comb-level dwell time, cross-frame movement, long-term spatial occupancy patterns, and changes in queen movement patterns that may reflect colony condition, thereby enabling new opportunities in both biological research and practical apiculture. At the same time, the current system is not intended for cell-level behavior analysis, such as confirming individual egg-laying events at specific wax cells, nor for fine-grained interaction analysis among multiple bees. In addition, although our experiments were conducted in a realistic full-size hive environment and show robustness under diverse environmental variations, they do not yet fully capture more realistic in-hive dynamics with denser worker-bee interference and continuously changing colony activity. Such effects are expected to manifest primarily as shifts in activation conditions rather than invalidate the underlying sensing mechanism, but evaluating SOPAR under these conditions remains an important direction for future work.

Proof of Theorem 3.1

Consider the varactor diode within the spiral resonator. At the initial stage of oscillation, the spiral resonator is excited to generate two frequency components: $f_{b}$ and $f_{c}$, where $f_{b}+f_{c}=f_{p}$. These three frequencies mix inside the diode. Denote $I_{b}$, $I_{c}$, and $I_{p}$ are the currents through the diode of these three frequency components and $V_{b}$, $V_{c}$, and $V_{p}$ as their voltages across the diode. Then, we have

(11) $$I_{c}+I_{b}+I_{p}=\frac{d\left[C(t)(V_{c}+V_{b}+V_{p})\right]}{dt}.$$

Both current and voltage terms in Eq. (11) can be written in their complex format for signal analysis, i.e.,

(12) $$V_{c}=R(v_{c}e^{j2\pi f_{c}t}),\;\;V_{b}=R(v_{b}e^{j2\pi f_{b}t}),\;\;V_{p}=R(v_{p}e^{j2\pi f_{p}t}),\;\;I_{c}=R(i_{c}e^{j2\pi f_{c}t}),\;\;I_{b}=R(i_{b}e^{j2\pi f_{b}t}),\;\;I_{p}=ℜ(i_{p}e^{j2\pi f_{p}t}),$$

where R(⋅) is the real part of a complex number, $i_{c}$, $i_{b}$, $i_{p}$, $v_{c}$, $v_{b}$ and $v_{p}$ are complex amplitudes of currents and voltages at $f_{c}$, $f_{b}$ and $f_{p}$, respectively.

Substituting Eq. (12) into Eq. (11) and with term elimination, the following impedance matrix can be obtained:

(13) $$\left[\begin{array}{c} v_{b}^{*}\\ v_{c} \end{array}\right]=\frac{1}{C_{0}2\pi f_{c}f_{b}(\phi^{2}-\lambda^{2}{|v_{p}|}^{2})}\left[\begin{array}{cc} j\phi^{2}f_{c} & j\lambda \phi v_{p}^{*}f_{b}\\ -j\lambda \phi v_{p}f_{c} & -j\phi^{2}f_{b} \end{array}\right]\left[\begin{array}{c} i_{b}^{*}\\ i_{c} \end{array}\right].$$

Using Kirchhoff’s Voltage Law to analyze the frequency component $f_{c}$, the equivalent voltage source $\xi_{c}$ of this frequency component can be expressed as

(14) $$\xi_{c}=v_{c}+i_{c}(R_{c}+j2\pi f_{c}L_{c})=\frac{-j\lambda \phi v_{p}f_{c}i_{b}^{*}-j\phi^{2}f_{b}i_{c}}{C_{0}2\pi f_{c}f_{b}(\phi^{2}-\lambda^{2}{|v_{p}|}^{2})}+i_{c}(R_{c}+j2\pi f_{c}L_{c})\approx \frac{-j\lambda \phi v_{p}i_{b}^{*}}{C_{0}2\pi f_{b}(\phi^{2}-\lambda^{2}{|v_{p}|}^{2})}+i_{c}(R_{c}+j2\pi f_{c}L_{c}),$$

where $R_{c}+j2\pi f_{c}L_{c}$ is the effective circuit impedance excluding the diode at frequency $f_{c}$.

Similarly, using Kirchhoff’s Voltage Law to analyze the frequency component $f_{b}$, the equivalent voltage source $\xi_{b}$ of this frequency component is obtained:

(15) $$\xi_{b}^{*}=v_{b}^{*}+i_{b}^{*}(R_{b}-j2\pi f_{b}L_{b})=\frac{j\lambda \phi v_{p}^{*}f_{b}i_{c}+j\phi^{2}f_{c}i_{b}^{*}}{C_{0}2\pi f_{c}f_{b}(\phi^{2}-\lambda^{2}{|v_{p}|}^{2})}+i_{b}^{*}(R_{b}-j2\pi f_{b}L_{b})\approx \frac{j\lambda \phi v_{p}^{*}i_{c}}{C_{0}2\pi f_{c}(\phi^{2}-\lambda^{2}{|v_{p}|}^{2})}+i_{b}^{*}(R_{b}-j2\pi f_{b}L_{b}),$$

where $R_{b}-j2\pi f_{b}L_{b}$ is the effective circuit impedance excluding the diode at frequency $f_{b}$.

To generate enlarged current flows for both frequency components, $i_{c}$ and $i_{b}^{*}$ need to be sufficiently large even when $\xi_{c}$ and $\xi_{b}^{*}$ are close to zero. Define $X_{c}=2\pi f_{c}L_{c}$ and $X_{b}=2\pi f_{b}L_{b}$. Then, the following requirement can be derived from Eq. (14) and Eq. (15).


(16) $$(R_{c}+jX_{c})(R_{b}-jX_{b})-\frac{\lambda^{2}\phi^{2}{|v_{p}|}^{2}f_{c}f_{b}}{C_{0}^{2}f_{c}^{2}f_{b}^{2}{\left(\phi^{2}-\lambda^{2}{|v_{p}|}^{2}\right)}^{2}}=0.$$

Define $T=\lambda |v_{p}|∕\phi$, Eq. (16) can be rewritten as

(17) $$R_{c}R_{b}+X_{c}X_{b}-\frac{T^{2}}{C_{0}^{2}4\pi^{2}f_{c}f_{b}{\left(1-T^{2}\right)}^{2}}=0.$$

Given that $T^{2}\ll 1$, Eq. (17) can be simplified to:

(18) $$T^{2}\approx C_{0}^{2}4\pi^{2}f_{c}f_{b}(R_{c}R_{b}+X_{c}X_{b}).$$

When the input and idler oscillation currents share the same resonance mode, the two frequency components converge to the same value, i.e., $f_{c}=f_{b}=f_{p}∕2=f_{0}$. For this degenerate current mode, the circuit has identical resistance ($R_{c}=R_{b}=R_{0}$) and reactance ($X_{c}=X_{b}=X_{0}$). Meanwhile, T reaches its minimum when $X_{c}=X_{b}=0$, corresponding to oscillation exactly at resonance. At this condition, Eq. (18) can be simplified to

(19) $$T^{2}\equiv \frac{\lambda^{2}{|v_{p}|}^{2}}{\phi^{2}}\approx {\left(C_{0}2\pi f_{0}R_{0}\right)}^{2}=\frac{1}{Q^{2}}.$$

For a resonator circuit with a quality factor Q, per Eq. (19), the required pumping voltage across the varactor diode is derived as:

(20) $$V_{p}^{⋆}\geq |v_{p}|\approx \frac{\phi }{\lambda Q}.$$

This completes the proof.

## Figures and Tables

**Table 1.** Comparison of RF-based systems for insect tracking in the literature.

Feature	Species	Batteryless?	Self-Interference present?	Tech & Band	Mass	Dimension	Detection Range	Tag-to-body mass ratio	Avg loc. error
Lorenzo-López *et al.* [25]	Queen bee	Yes	Yes	UHF RFID; 868 MHz	25 mg	3.09×2.61×0.25 mm	N/A	10 %	N/A
Souza *et al.* [11]	Apis mellifera	Yes	Yes	UHF RFID; 915 MHz	5.4 mg	2.5×2.5×0.4 mm	N/A	N/A	N/A
Barlow *et al.* [4]	Bumblebee	Yes	Yes	UHF RFID; 915 MHz	81 mg	7×2 mm	0.75 m	49 %	N/A
Alburaki *et al.* [2]	Apis mellifera	Yes	Yes	UHF RFID; 915 MHz	5 mg	2.5×2.5×0.4 mm	1.5 cm	5 %	N/A
Track-a-Forager [47]	Apis mellifera	Yes	No	HF RFID; 13.56 MHz	5 mg	5×1.7×0.5 mm	mm-level (<1 cm)	N/A	N/A
*μ*Locate [35]	N/A	No	N/A	Backscatter; Multi-band	N/A	11.8×7.5×2.1 mm	60 m	N/A	1.45 m
Living IoT [16]	Bumblebees	No	Yes	Backscatter; 915 MHz	102 mg	6.4×6.1 mm^2^	80 m	N/A	1.90 m
Maggiora *et al.* [30]	Vespa velutina	Yes	No	Harmonic radar; TX/RX: 9.4/18.8 GHz	15 mg	12×4×3 mm	500 m	N/A	7 m
Woodgate *et al.* [55]	Bombus	Yes	No	Harmonic radar; TX/RX: 9.4/18.8 GHz	15 mg	16 mm	800 m	10 %	2 m
Milanesio *et al.* [33]	Vespa velutina	Yes	No	Harmonic radar; TX/RX: 9.4/18.8 GHz	12 mg	16×2.5×0.25 mm	125 m	N/A	1.50 m
Miller *et al.* [34]	Melon fly	Yes	No	Harmonic radar	0.8 mg	40 mm	5 m	5 %	2 m
Airdrop Sensors [15]	Manduca sexta	No	No	BLE; 2.4 GHz	203 mg	N/A	1,000 m	N/A	≈1.3°
Huang *et al.* [14]	Mecynorhina	No	No	BLE; 2.4 GHz	1,580 mg	N/A	160 m	N/A	N/A
Jiang *et al.* [17]	Mecynorhina	No	No	Bluetooth; 2.4 GHz	523 mg	N/A	160 m	N/A	N/A
Shearwood *et al.* [41]	Honey-bee	Yes	No	RF beacon; 5.8 GHz	N/A	31.8×1×0.39 mm	N/A	120 %	± 5°
mSAIL [20]	Monarch butterfly	No	No	Active radio; 915 MHz	62 mg	8×8×2.6 mm	LOS/NLOS: 150/494 m	12.4 %	21.4/44.5 km
Dedic *et al.* [12]	400mg Bumblebee	Yes	N/A	FMCW; 77 GHz	50 mg	15×2 mm	1.77 m	13 %	0.30 m
**SOPAR (This work)**	**Honeybee**	**Yes**	**No**	**Backscatter; TX/RX: 1344/672 MHz**	**10 mg**	**3.7 mm (diameter)**	**1 m**	**4.8 %**	**3.7 cm (median)**

## CCS Concepts:

• Human-centered computing → Ubiquitous and mobile computing systems and tools; • Hardware → Sensor devices and platforms.
